# Antimicrobial perceptions and stewardship practices among community pharmacy dispensers in Nepal

**DOI:** 10.1017/ash.2025.10158

**Published:** 2025-10-14

**Authors:** Bikalpa Shrestha, Sweta Shrestha, Upasana Acharya

**Affiliations:** Department of Pharmacy, Kathmandu University, Dhulikhel, Kavre, Nepal

## Abstract

**Objective:**

This study assessed the perception and practice regarding Antimicrobial Stewardship (AMS) and knowledge about antibiotics among Community Pharmacy Dispensers (CPD) in selected municipalities of Kavrepalanchowk district, Nepal.

**Design:**

A cross-sectional study was conducted among CPD of the community pharmacies of Banepa, Dhulikhel, and Panauti in Kavrepalanchowk district, Nepal.

**Methods:**

A structured self-administered questionnaire was administered to 58 CPD selected through census sampling technique. The questionnaire comprised of questions assessing the knowledge of antibiotics, perception, and practice regarding AMS. A bivariate analysis was done to determine association between demographic variables and dependent variables.

**Results:**

Majority of respondents (60.3%) had medium level of knowledge regarding antibiotics, 46.6% of the respondents had low practice scores, and 50% of the respondents had positive perceptions of AMS. A positive correlations of knowledge with perception (*p* = 0.0001) and practice (*p* = 0.019) was seen. Education level had a significant association with knowledge levels (*p* = 0.035) and perception about AMS (*p* = 0.043). A significant association between gender with AMS practice was also observed (*p* = 0.002).

**Conclusion:**

The practice of AMS in community pharmacies is low despite of medium level of knowledge on antibiotics among the community pharmacy dispensers. Establishing AMS protocols specific to community pharmacies in Nepal can lead to standardized practices and improve adherence to AMS principles.

## Introduction

Antimicrobial resistance (AMR) has become a critical global public health threat, contributing to increased morbidity, mortality, and healthcare burdens.^1,2^ The consequences of AMR include prolonged hospitalizations, higher treatment costs, and adverse effects from excessive antibiotic use.^3^ The World Health Organization (WHO) has launched initiatives to promote rational antibiotic prescribing.^4^ However, Nepal continues to face alarming AMR rates, with resistance exceeding 50% for pathogens like *Escherichia coli, Staphylococcus aureus, and Pseudomonas aeruginosa*.^5–7^ Unnecessary antibiotic prescriptions, self-medication, and lack of susceptibility testing further exacerbate the problem.^5^ In Nepal, antibiotics are commonly sold over-the-counter (OTC) without prescriptions, despite legal restrictions. This widespread availability promotes self-medication and contributes significantly to AMR. Recognizing the urgency, the Department of Drug Administration (DDA) recently imposed a ban on the OTC sale of six critical reserve antibiotics—including meropenem, vancomycin, piperacillin + tazobactam, polymyxin B, linezolid, and colistin—restricting their distribution to hospital pharmacies only. This important regulatory step aims to reduce misuse of last-resort antibiotics and align Nepal with global AMR containment efforts.^8^ However, challenges remain in enforcement and monitoring, especially in community pharmacy settings. Nepal’s National Action Plan (NAP) on AMR (2021–2026) aligns with the WHO’s Global Action Plan (GAP), emphasizing AMS to optimize antibiotic use.^9^ Community pharmacists play a crucial role in AMS, yet their engagement remains limited due to insufficient training and collaboration.^10,11^ In several countries, community pharmacists have become integral members of antimicrobial stewardship (AMS) programs by actively engaging in activities such as prescription screening for appropriateness, patient counseling on antimicrobial use, monitoring for adverse effects, and contributing to antimicrobial use surveillance systems. For example, in the United Kingdom and Australia, community pharmacists routinely review antibiotic prescriptions against national guidelines and provide targeted education to patients on dosage, duration, and adherence.^12^ Adopting similar roles in the Nepalese context—supported by targeted AMS training, regulatory enforcement, and structured collaboration with prescribers—could significantly enhance rational antibiotic use and help curb the growing AMR problem. Studies indicate that antibiotics are frequently dispensed without prescriptions in Nepal, worsening resistance.^13^ CPDs who dispense antibiotics in community pharmacies in Nepal, typically fall into three categories: Pharmacists—Hold a Bachelor’s degree in Pharmacy (BPharm), Pharmacy Assistants—Have completed a 3-year Diploma in Pharmacy, Orientation holder—Have received limited or informal training but often manage or assist in pharmacy settings.^14^ It is thus crucial to understand CPD’s perceptions and current practices regarding ASP before incorporating them in the development and implementation of AMS programs.

## Methods

### Study design and study site

A descriptive, cross-sectional study was conducted over a period of three months from April to June 2024 in community pharmacies of Banepa, Dhulikhel, and Panauti municipalities. A census sampling approach was used to include all available CPD from the selected municipalities of Kavrepalanchok district. A complete list of registered community pharmacies was obtained from DDA, and each pharmacy was visited. One CPD from each pharmacy, who was present during data collection was included in the study. Total of 58 CPD were enrolled in this study.

### Study participants

One Nepal Pharmacy Council (NPC) certified CPD was selected from each DDA registered community pharmacy located in Banepa, Dhulikhel, and Panauti.

### Data collection tool

A validated structured questionnaire consisting of 31 items was used after seeking permission from the author.^10^ The questionnaire was divided into four sections. Section I comprised of the socio-demographic, Section II and Section III comprised of 8 Likert type statements on antibiotics and perceptions of AMS. A 5-point Likert scale was used where 1 denotes strongly disagree, 2 disagree, 3 neutral, 4 agree, and 5 strongly agree. Section IV consisted of 11 statements on practices of participants on AMS rated with a 5-point Likert scale, score ranging from 1 for never, 2 for rarely, 3 for occasionally, 4 for often, and 5 for always. Each correct statement agreed upon by the participants resulted in a higher score. Failure to agree on the right statements resulted in a lower score. Likewise, a higher score was granted to the participants who disagreed with negative questions and vice versa. Scoring of knowledge and practice was done as follows: score ≤50% was denoted “low,” 50–75% “medium,” and ≥75% “High.” Outcomes regarding perception was assigned as “negative,” “fair,” and “positive” for those scoring ≤50%, 50–75%, and ≥75%, respectively.^15^

### Validation of data collection tool

A pilot study was conducted among 9 CPD of the community pharmacies located in Bhaktapur. The questionnaire was administered to them to ensure its comprehensiveness, appropriateness, and simplicity. A Cronbach’s alpha of perception, practice, and knowledge (0.705, 0.705, and 0.795) was obtained. The pilot study data was not incorporated into the final analysis.

### Data collection

After obtaining approval from Institutional Review Committee of Kathmandu University School of Medical Sciences (Approval no. 50/24), the data was collected from April 2024 to June 2024. One CPD from each registered pharmacies located at Banepa, Dhulikhel, and Panauti was selected. After explaining the research objective and obtaining a written consent from the participants, the self-administered questionnaire was provided to them.

### Statistical analysis

Epi data version 3.1 was used for data entry and then exported to Statistical Package for Social Sciences (SPSS) version 16. A *p* value <0.05 was considered statistically significant. A descriptive analysis was performed using frequencies and percentages. Pearson *χ* ^2^ test (*χ* ^2^) for independence was used to determine the association of socio-demographic variables with perception, knowledge, and practice. Binary logistic regression analysis was done to identify the determinant variables of knowledge, perception, and practice.

## Results

Demographics of the CPDs are presented in Table 1. Most CPDs demonstrated good understanding of the use of antibiotics for treating bacterial infections. However, misconceptions persisted—approximately one-sixth of the participants believed antibiotics could treat viral illnesses, and some also misunderstood its’ role in pain or inflammation. Few participants also opined on prematurely stopping antibiotics once symptoms improved (Table 2).

**Table 1.** Demographic characteristics of community pharmacy dispensers

Variable	Category	Frequency (%) (*n* = 58)
Sex	Male	23 (39.7)
	Female	35 (60.3)
Age (Years)	<20	3 (5.2)
	20–29	32 (55.2)
	30–39	12 (20.7)
	40–49	8 (13.8)
	≥50	3 (5.2)
Educational level	<Bachelor in pharmacy	41 (70.7)
	≥Bachelor in pharmacy	17 (29.3)
Practice years	<5 years	35 (60.3)
	≥5 years	23 (39.7)

**Table 2.** Community pharmacy dispensers’ knowledge about antibiotics

**Variables**	**Response (*n* = 58)**				
	**SD** ***N* (%)**	**D** ***N* (%)**	**N** ***N* (%)**	**A** ***N* (%)**	**SA** ***N* (%)**
Antibiotics are useful for bacterial infections (eg, tuberculosis)	1 (1.7)	3 (5.2)	3 (5.2)	27 (46.6)	24 (41.4)
Antibiotics are useful for viral infections (eg, flu)	13 (22.4)	24 (41.4)	12 (20.7)	5 (8.6)	4 (6.9)
Antibiotics are indicated to reduce any kind of pain and inflammation.	6 (10.3)	24 (41.4)	9 (15.5)	13 (22.4)	6 (10.3)
Antibiotics can kill “normal flora” present in our body.	4 (6.9)	3 (5.2)	12 (20.7)	28 (48.3)	11 (19.0)
Antibiotics can cause secondary infections after killing normal flora present in our body.	0 (0)	7 (12.1)	10 (17.2)	33 (56.9)	8 (13.8)
Antibiotics can cause allergic reactions.	1 (1.7)	2 (3.4)	4 (6.9)	33 (56.9)	18 (31.0)
Misuse of antibiotics can lead to a loss of sensitivity of an antibiotic to a specific pathogen.	2 (3.4)	0 (0)	5 (8.6)	33 (56.9)	18 (31.0)
If symptoms improve before the full antibiotic course is completed, you can stop taking it.	31 (53.4)	15 (25.9)	0 (0)	9 (15.5)	3 (5.2)

SA, strongly agree; A, agree; N, neutral; DA, disagree.

Perceptions toward AMS were largely positive. Over 80% agreed AMS improves patient care and more than one-half of the participants recommended its’ integration at the community pharmacy level. However, less than one-third believed AMS is solely the physician’s responsibility (Table 3).

**Table 3.** Respondents’ perceptions of antimicrobial stewardship program

Variables	**Response (*n* = 58)**				
	SD *N* (%)	D *N* (%)	N *N* (%)	A *N* (%)	SA *N* (%)
AMS programs improve patient care.	2 (3.4)	1 (1.7)	6 (10.3)	35 (60.3)	14 (24.1)
AMS should be incorporated at the community- pharmacy level.	1 (1.7)	2 (3.4)	3 (5.2)	40 (69.0)	12 (20.7)
AMS program reduce problems of anti-microbial resistance	0 (0.0)	2 (3.4)	7 (12.1)	32 (55.2)	17 (29.3)
Adequate training on antimicrobial use should be provided to community pharmacists.	1 (1.7)	6 (10.3)	0 (0.0)	29 (50)	22 (37.9)
Relevant conferences, workshops, and other educational activity are required to be attended by community pharmacists to enhance understanding of antimicrobial stewardship.	1 (1.7)	3 (5.2)	0 (0.0)	26 (44.8)	28 (48.3)
Individual efforts at antimicrobial stewardship have minimal impact on the antimicrobial-resistance problem.	3 (5.2)	16 (27.6)	7 (12.1)	27 (46.6)	5 (8.6)
I think that prescribing physicians are the only professionals who need to understand antimicrobial stewardship.	20 (34.5)	19 (32.8)	1 (1.7)	12 (20.7)	6 (10.3)
Pharmacists have a responsibility to take a prominent role in antimicrobial stewardship and infection-control programs in the health system.	1 (1.7)	0 (0.0)	5 (8.6)	35 (60.3)	17 (29.3)

SA, strongly agree; A, agree; N, neutral; DA, disagree.

Practices were inconsistent. While one-third always dispensed antibiotics with complete clinical information, over 50% admitted to dispensing antibiotics without prescriptions. Over one-third also dispensed antibiotics exceeding the prescribed quantities on patient’s request. Engagement in AMS activities like patient education, collaboration, and infection control was moderate, highlighting gaps between awareness and practice (Table 4).

**Table 4.** Community pharmacy dispensers’ practices of AMS

Variables	**Response (n = 58)**				
	Never *N* (%)	Rarely *N* (%)	Occasionally N (%)	Often *N* (%)	Always *N* (%)
I dispense antimicrobial on prescription with complete clinical information.	6 (10.3)	11 (19)	12 (20.7)	10 (17.2)	19 (32.8)
I dispense antimicrobials without a prescription.	12 (20.7)	10 (17.2)	16 (27.6)	15 (25.9)	5 (8.6)
I dispense antimicrobials for durations longer than prescribed by the physician on patient request.	24 (41.4)	4 (6.9)	6 (10.3)	18 (31.0)	6 (10.3)
I screen antimicrobial prescriptions in accordance with local guidelines before dispensing.	17 (29.3)	13 (22.4)	17 (29.3)	6 (10.3)	5 (8.6)
I collaborate with other health professionals on infection control and antimicrobial stewardship.	22 (37.9)	10 (17.2)	6 (10.3)	9 (15.5)	11 (19)
I communicate with prescribers if I am unsure about the appropriateness of an antibiotic prescription.	10 (17.2)	10 (17.2)	10 (17.2)	10 (17.2)	18 (31)
I have sought additional clinical information (eg, drug interaction, adverse drug reactions, allergy) before deciding to dispense the antibiotic prescribed.	10 (17.2)	14 (24.1)	9 (15.5)	8 (13.8)	17 (29.3)
I take part in antimicrobial-awareness campaigns to promote the optimal use of antimicrobials.	8 (13.8)	21 (36.2)	13 (22.4)	5 (8.6)	11 (19.0)
I educate patients on the use of antimicrobials and resistance-related issues.	6 (10.3)	13 (22.4)	12 (20.7)	8 (13.8)	19 (32.8)
I make efforts to prevent or reduce the transmission of infections within the community.	5 (8.6)	16 (27.6)	8 (13.8)	10 (17.2)	19 (32.8)
I ask patients about their knowledge of prescribed antimicrobials and their usage.	12 (20.7)	14 (24.1)	13 (22.4)	8 (13.8)	11 (19.0)

Majority of respondents, (60.3%), had a medium level of knowledge regarding antibiotics whereas 46.6% had low practice scores [Supplementary Table I]. Half of the respondents (50%) had a positive perception regarding AMS [Supplementary Table II]. A significant positive correlation was observed between perception and knowledge scores (*P* < 0.01) and practice and knowledge scores (*p* < 0.05) [Supplementary Table III].

A significant association was found between education level and knowledge about antibiotics (*P* < 0.05). Binary logistic regression analysis showed that participants with education below bachelor’s degree were 3.45 times more likely to be less knowledgeable than participants with Bachelors’ degree or higher qualifications (AOR: 3.452, 95% CI: 1.064–11.203, *P* < .001). [Table 5]

**Table 5.** Association of demographic with knowledge

**Variables**	**Low to medium** ***N* (%)**	**High** ***N* (%)**	***P* value**	**Adjusted Odds Ratio (AOR) (95% Confidence Interval)**
Sex				
Male	14 (60.9)	9 (39.1)	0.879	
Female	22 (62.9)	13 (37.1)		
Age				
<30 years	24 (70.6)	10 (29.4)	0.111	
≥30 years	12 (50)	12 (50)		
Education level				
<Bachelor in Pharmacy	29 (70.7)	12 (29.3)	0.035^*^	3.452 (1.064,11.203)
≥Bachelor in Pharmacy	7 (41.2)	10 (58.8)		
Practice years				
<5 years	24 (68.6)	11 (31.4)	0.208	
≥5 years	12 (52.2)	11 (47.8)		

* indicate statistically significance at p value<.05.

A significant association was also observed between education level and perception about AMS with *P* < 0.05. Participants with less than a bachelor’s degree in pharmacy were 3.39 times more likely to have low to medium perception on AMS than a bachelor’s degree or higher (AOR: 3.388, 95% CI: (1.006–11.411), *P* < 0.05). [Table 6]

**Table 6.** Association of demographic with perception

Variables	Low to medium *N* (%)	High *N* (%)	*P* value	Adjusted Odds Ratio (AOR) (95% Confidence Interval)
Sex				
Male	11 (47.8)	12 (52.2)	0.788	
Female	18 (51.4)	17 (48.6)		
Age				
<30 years	18 (52.9)	16 (47.1)	0.594	
≥30 years	11 (45.8)	13 (54.2)		
Education level				
<Bachelor in Pharmacy	24 (58.5)	17 (41.5)	0.043^*^	3.388 (1.006,11.411)
≥Bachelor in Pharmacy	5 (29.4)	12 (70.6)		
Practice years				
<5 years	16 (45.7)	19 (54.3)	0.421	
≥5 years	13 (56.5)	10 (43.5)		

* indicate statistically significance at *p* value < 0.05.

A significant association was found between gender and AMS practice (*P* < 0.05). Male participants were 0.1 times less likely to have low to medium AMS practice compared to female participants (AOR: 0.153, 95% CI: 0.044–0.534, *P* < 0.05). [Table 7]

**Table 7.** Association of demographic with practice

Variables	Low to medium *N* (%)	High *N* (%)	*P* value	Adjusted Odds Ratio (AOR) (95% Confidence Interval)
Sex				
Male	11 (47.8)	12 (52.2)	0.002^*^	0.153 (0.044,0.534)
Female	30 (85.7)	5 (14.3)		
Age				
<30 years	27 (79.4)	7 (20.6)	0.082	
≥30 years	14 (58.3)	10 (41.7)		
Education level				
<Bachelor in Pharmacy	32 (78)	9 (22)	0.056	
≥Bachelor in Pharmacy	9 (52.9)	8 (47.1)		
Practice years				
<5 years	28 (80)	7 (20)	0.055	
≥5 years	13 (56.5)	10 (43.5)		

## Discussion

This study evaluated the knowledge, perceptions, and practices related to AMS among CPDs in three municipalities of Kavrepalanchok, Nepal. Our findings reveal that while general awareness of antibiotic use is present, substantial gaps persist in understanding of key areas such as ineffectiveness of antibiotics against viral infections, the risks of incomplete treatment courses and misconception about use of antibiotics for treating pain and inflammation. These knowledge gaps are consistent with trends reported globally, although the exact prevalence varies across regions.^10,16–18^ For instance, a high level of knowledge regarding antibiotics was seen only among 37.9% of our respondents which is lower than the 70.6% reported in a study in Nigeria.^16^ Similarly, although misconceptions about the role of antibiotics in treating viral infections still existed, the level of unawareness was lower compared to findings from studies conducted in Italy and Sri Lanka. Likewise, although only a smaller proportion of our respondents were unaware about the link between antibiotic misuse and resistance as compared to other studies,^10,17^ such misconceptions still persisted. CPD’s lack of knowledge on crucial aspects such as its’ ineffectiveness against viruses and hence its’ inappropriateness in common cold, can lead to unnecessary use of antibiotics thus fueling AMR.^19^ This issue can further be aggravated in situations where dispensers are ignorant of the risk of AMR due to misuse of antibiotics.^20^ This is more worrisome in countries like Nepal where antibiotics are dispensed without prescription from community pharmacies.^21^ However in context of Nepal, there has been a noticeable increase in AMR-focused trainings and workshops in Nepal. Awareness campaign like a global campaign, World Antimicrobial Awareness Week (WAAW) is conducted from 18–24 November every year in Nepal.^22^ Over 100 participants were trained in March 2025 on the WHO AMC to improve Nepal’s antimicrobial use surveillance under GLASS-AMC (Global AMR and Use Surveillance System- Antimicrobial Consumption). Such initiatives may have probably accounted for the relatively lower percentage of unawareness in our study compared to other studies.

Encouragingly, most CPDs exhibited positive perceptions toward AMS and believed in its’ strength in improving patient care, which aligns with the findings from other countries like Pakistan, Malaysia, and Ethiopia.^10,11,23^ However, only about half of the respondents recognized the importance of integrating AMS programs within community pharmacy settings. Literatures report varied data on this regard.^10^ This variation may reflect differing levels of AMS program implementation and awareness campaigns across countries. Majority of our respondents also agreed that community pharmacists have a prominent role in AMS program in healthcare settings which align with the findings from a systematic review.^23^ ASPs implementation demands team efforts, and multidisciplinary collaboration from many healthcare professionals.^24^ Approximately 46.6% participants were in agreement to this notion. In consensus with our findings, a study revealed that 55% of the respondents were in support for the introduction of a specialized multidisciplinary team to provide guidance on antimicrobial prescribing.^25^ A shared decision making enhances the collaboration between pharmacists and other healthcare professionals.^26^ Community pharmacist contribute to surveillance systems by reporting antibiotic sales and dispensing data, helping in the detection of resistance trends and informing targeted intervention.^12^ Without team collaboration, pharmacy dispenser may not have major impact on the processes of enhancing quality of antibiotic prescription. Inter-professional areas need a lot of emphasis to ensure the effectiveness of antibiotic stewardship roles in community pharmacy settings of Nepal.

This study also highlights a concerning disconnect between knowledge/perception and actual AMS-related practices. A significant number of CPDs still dispense antibiotics without prescriptions. These practices echo findings from other LMICs and underscore ongoing regulatory and systemic barriers. ^10,11,23,27^ Dispensing antibiotics without prescription and use of antibiotics for longer duration have been quoted as factors promoting the emergence of an antibiotic-resistant organism.^28,29^ Irrespective of majority of the respondents being aware of the misuse of antibiotics as a possible cause leading to emergence of AMR, its’ translation into practice was not evident. This might be due to the weak regulation and enforcement of the Drug Act 1978 in Nepal, which categorizes antibiotics under “Prescription Only Medication”.^30^ However, inadequate surveillance by DDA has led to extensive over the counter sales of antibiotics.^31^

Similarly, CPDs’ collaboration with other healthcare professionals was found to be limited. A small proportion of CPDs collaborated with other healthcare professionals for infection control and AMS. This collaboration primarily included activities such as seeking guidance from prescribers on the appropriateness of antibiotic prescriptions, sharing patient treatment histories to optimize antibiotic use, and participating in discussions related to infection control. This highlights the existing crevices in inter-professional collaboration between CPD and prescribers in our setting. Such collaborations have been associated with enhanced patient care.^32^ Enhanced communication between healthcare professionals could significantly aid in optimizing the antibiotic use. Poor inter-professional collaborations further highlight a missed opportunity to optimize antibiotic use. In contrast, studies in countries with stronger stewardship infrastructure report higher engagement and teamwork between pharmacists and prescribers.^11,23,33^ Similarly, lesser percentage of the respondents considered educating patients on use of antimicrobials and resistance. This underscores the necessity to encourage pharmacists to participate in antimicrobials awareness programs and promote public and professional awareness on the significance of rational use of antibiotics.

Education level was significantly associated with both knowledge and perception, consistent with global findings.^11,25,34^ CPDs with higher academic qualifications are more likely to understand and implement AMS principles, emphasizing the need for formalized AMS training during pharmacy education.

In Nepal, where antibiotics are often dispensed without a prescription despite regulatory restrictions,^30,31^ strengthening surveillance and regulatory enforcement is essential. Additionally, targeted AMS training and integration of CPDs into national stewardship efforts could significantly curb inappropriate antibiotic use and AMR.

Community pharmacy dispensers in this study demonstrated moderate knowledge and perception of AMS, but their actual practices reveal significant room for improvement. Strengthening education, regulatory oversight, and inter-professional collaboration is essential to closing the gap between knowledge and practice. Future research should explore specific barriers to AMS adherence among CPDs and inform the design of practical, community-level stewardship interventions.

## Supporting information

10.1017/ash.2025.10158.sm001 Shrestha et al. supplementary material 1 Shrestha et al. supplementary material

10.1017/ash.2025.10158.sm002 Shrestha et al. supplementary material 2 Shrestha et al. supplementary material

10.1017/ash.2025.10158.sm003 Shrestha et al. supplementary material 3 Shrestha et al. supplementary material

## Supplementary material

The supplementary material for this article can be found at https://doi.org/10.1017/ash.2025.10158.
