# Supplementary material for: Antimicrobial perceptions and stewardship practices among community pharmacy dispensers in Nepal
Source: Antimicrob Steward Healthc Epidemiol. 2025 Oct 14;5(1):e259. doi: 10.1017/ash.2025.10158 (PMC12538339; doi:10.1017/ash.2025.10158)
Supplement: Shrestha et al. supplementary material 1 — Shrestha et al. supplementary material [file S2732494X25101587sup001.docx]

**Supplementary Table III: Correlation between Perception, Practice, and Knowledge Scores**

|  |  |  |  |
| --- | --- | --- | --- |
| Variables | Practice score  p value | Perception  p value | Knowledge  p value |
| Practice |  | 0.397 | 0.019* |
| Perception | 0.397 |  | 0.0001* |
| Knowledge | 0.019* | 0.0001* |  |
