# Supplementary material for: Antimicrobial perceptions and stewardship practices among community pharmacy dispensers in Nepal
Source: Antimicrob Steward Healthc Epidemiol. 2025 Oct 14;5(1):e259. doi: 10.1017/ash.2025.10158 (PMC12538339; doi:10.1017/ash.2025.10158)
Supplement: Shrestha et al. supplementary material 2 — Shrestha et al. supplementary material [file S2732494X25101587sup002.docx]

**Supplementary Table I: Distribution of Knowledge, Practice Scores among Community Pharmacy Dispensers**

| **Score** | **Low**  **N(%)** | **Medium**  **N (%)** | **High**  **N(%)** |
| --- | --- | --- | --- |
| Knowledge Score | 1(1.7) | 35(60.3) | 22(37.9) |
| Practice Score | 27(46.6) | 14(24.1) | 17(29.3) |
