# Supplementary material for: Antimicrobial perceptions and stewardship practices among community pharmacy dispensers in Nepal
Source: Antimicrob Steward Healthc Epidemiol. 2025 Oct 14;5(1):e259. doi: 10.1017/ash.2025.10158 (PMC12538339; doi:10.1017/ash.2025.10158)
Supplement: Shrestha et al. supplementary material 3 — Shrestha et al. supplementary material [file S2732494X25101587sup003.docx]

**Supplementary Table II: Perception Scores among Community Pharmacy Dispensers**

| **Score** | **Fair**  **N (%)** | **Positive**  **N (%)** | **Negative**  **N (%)** |
| --- | --- | --- | --- |
| Perception Score | 29(50.0) | 29(50.0) | 0(0.0) |
